# Supplementary material for: Complexome profiling of the Chlamydomonas psb28 mutant reveals TEF5 as an early PSII assembly factor
Source: Plant Cell. 2025 Mar 17;37(6):koaf055. doi: 10.1093/plcell/koaf055 (PMC12164590; doi:10.1093/plcell/koaf055)
Supplement: koaf055_Supplementary_Data [file koaf055_supplementary_data.zip › psb28_interactive.zip!/plots/intensity/qualityPlots/peptides/NUOB22_Cre11.g467668.t1.1.html]

### Cre11.g467668.t1.1

Trivial name: NUOB22  
SeqCoverage [median]: 39.30  
Mass: 13.93 kDa  
  
 

| Sequence | MissedCleavage | Mass [Da] | wtIntensity | muIntensity | Score |
| --- | --- | --- | --- | --- | --- |
| ALTDREEIR | 1 | 1101.58 | 793.07 | 7779.38 | 57.96 |
| ALTDREEIRK | 2 | 1229.67 | 1582.81 | 2324.24 | 68.89 |
| ASEVVLALPHK | 0 | 1162.67 | 21329.65 | 36595.46 | 200.05 |
| ASEVVLALPHKLR | 1 | 1431.86 | 395.14 | 3118.81 | 86.31 |
| AYALRQEFEDSK | 1 | 1455.70 | 8354.55 | 6204.84 | 67.46 |
| KKLEHGEELLAR | 2 | 1421.80 | 0.00 | 1313.37 | 81.92 |
| KLEHGEELLAR | 1 | 1293.70 | 5984.57 | 1783.96 | 103.91 |
| QEFEDSK | 0 | 881.38 | 1740.77 | 614.25 | 113.70 |
|  |  |  | 40180.6 | 59734.3 |  |
